# COVID-19 Seroprevalence and Active Infection in an Asymptomatic Population

**DOI:** 10.3389/fmed.2021.749732

**Published:** 2021-09-13

**Authors:** Amy M. E. Breedon, Roland J. Saldanha, Richard L. Salisbury, David E. Metzger, Michael P. Werry, Craig J. McPherson, Adam P. Irvin, Christina M. Davis, Charles A. Bogner, Amber M. Braddock, Charles E. Salter, Claude C. Grigsby, Corey R. Hart, Heather A. Pangburn

**Affiliations:** ^1^Air Force Research Laboratory, 711th Human Performance Wing, Wright-Patterson Air Force Base, OH, United States; ^2^UES, Inc., Integrative Health & Performance Sciences Division, Dayton, OH, United States

**Keywords:** COVID-19, SARS-CoV-2, saliva, testing, seroprevalance, antibodies, asymptomatic

## Abstract

In response to the COVID-19 pandemic, immediate and scalable testing solutions are needed to direct return to full capacity planning in the general public and across the Department of Defense (DoD). To fully understand the extent to which a population has been affected by COVID-19, active monitoring approaches require an estimation of overall seroprevalence in addition to accurate, affordable, and rapid tests to detect current SARS-CoV-2 infection. In this study, researchers in the Air Force Research Laboratory's 711th Human Performance Wing, Airman Systems Directorate evaluated the performance of various testing methods for the detection of SARS-CoV-2 antibodies and viral RNA in asymptomatic adults working at Wright-Patterson Air Force Base and the surrounding area during the period of 23 July 2020–23 Oct 2020. Altogether, there was a seroprevalance of 3.09% and an active infection rate of 0.5% (determined *via* the testing of saliva samples) amongst individuals tested, both of which were comparable to local and national averages at the time. This work also presents technical and non-technical assessments of various testing strategies as compared to the gold standard approaches (e.g., lateral flow assays vs. ELISA and RT-LAMP vs. RT-PCR) in order to explore orthogonal supply chains and fieldability. Exploration and validation of multiple testing strategies will allow the DoD and other workforces to make informed responses to COVID-19 and future pandemics.

## Introduction

Severe acute respiratory syndrome coronavirus-2 (SARS-CoV-2) has infected over 33 million individuals in the United States alone as of 2 June 2021, resulting in almost 600,000 deaths (1). With over 295,000 cases reported in U.S. Department of Defense (DoD) personnel (2), the novel coronavirus disease-2019 (COVID-19) pandemic has altered the DoD's ability to work at full capacity and has highlighted readiness concerns for the U.S. military as a whole. Rapid and sensitive testing is vital to identifying potential SARS-CoV-2 outbreaks in order to maintain force readiness and to quantify the epidemiological impact of this and future pandemics.

Widespread testing is critical to allowing a return to full capacity in order to effectively support the overall mission while ensuring personnel safety, especially for those working in close quarters. Notably, the prevalence of asymptomatic or pre-symptomatic infection and transmission means that testing individuals only when symptoms arise can result in unnecessary spread of the disease (3–9). To understand the extent to which a workforce population has been or is being affected during this and any future pandemic, active monitoring approaches require both an estimation of seroprevalence in asymptomatic individuals as well as rapid, accurate, and affordable molecular testing to detect current infections (10).

Serological testing for the presence of viral antibodies in blood and serum can identify individuals with past exposure or infection. The presence and antibody isotype of SARS-CoV-2 reactive antibodies can be determined using an enzyme-linked immunosorbent assay (ELISA), the current “gold standard” in serology testing. This method has previously been optimized for SARS-CoV-2 antibody detection by Stadlbauer et al. (11) and Klumpp-Thomas et al. (12), with the latter reporting >99% for both specificity and sensitivity for SARS-CoV-2 antibody detection of Immunoglobulin M (IgM), Immunoglobulin G (IgG), and Immunoglobulin A (IgA) antibodies. The temporal antibody response to SARS-CoV-2 infection was recently analyzed in a systematic review of 150 studies (13). Briefly, IgM antibodies are generally detectable around 1 week after the initial onset of symptoms, peak around 2–5 weeks after onset, and decrease below detectable limits by 7–8 weeks post onset. In contrast, IgG levels rises to detectable levels ~2 weeks post symptom onset, peak around 3–7 weeks post symptom onset, and can remain elevated for an unknown time, although often reported as declining beyond 8 weeks. Finally, IgA antibodies peak around 2–3 weeks post symptom onset, however their pattern is less studied and understood. Early detection and monitoring of SARS-CoV-2 antibody levels in asymptomatic populations can provide a better understanding of exposure level and immune response, allowing for population-level estimates to inform safe return to full capacity decisions.

In contrast to serological testing, molecular testing is essential for active infection surveillance and screening, including monitoring disease prevalence, identifying different strains and mutations, and assessing the current infection rates within a large workplace population. The current gold standard molecular test for active SARS-CoV-2 infection is reverse transcription polymerase chain reaction (RT-PCR), often conducted on nasopharyngeal (NP) swabs (14–16). NP swabs are invasive, require special personal protective equipment (PPE) and trained expertise for sample collection, and have been prone to supply chain shortages thereby limiting the capacity of testing. Saliva has been shown to be a robust alternative biofluid that provides comparable results to NP swabs and is more easily collected, reducing the need for trained technicians and minimizing PPE requirements (17–22). Another alternative to the current standard is using a different detection assay, for example reverse transcription loop-mediated isothermal amplification (RT-LAMP) (14, 15, 23–26). RT-LAMP, which has previously been used to identify other viruses including influenza strains (27), has been documented as a simple, fast, and cost-effective method that uses alternate enzymes and equipment than those used for RT-PCR, mitigating supply chain constraints. In addition, the simplicity of the assay requirements enables the use of less advanced laboratory equipment and deployment to more austere environments to provide rapid results (28).

**Figure 1:**
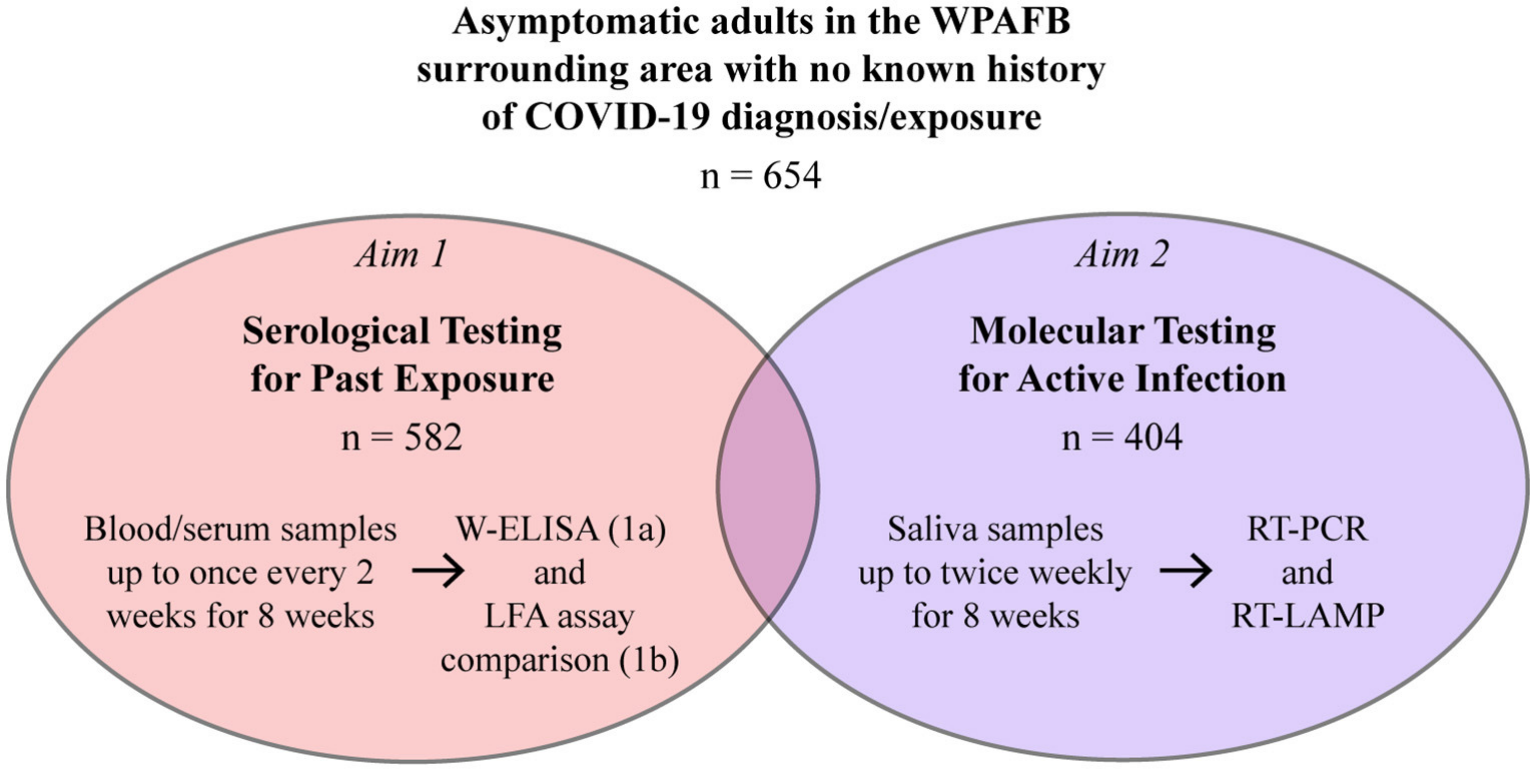
Overview of the study. Participants enrolled in the serosurvey (Aim 1a or Aims 1a and 1b) and/or the molecular survey (Aim 2).

The present study investigated evidence-based solutions for widespread, rapid, and accurate testing in a large workforce population. Specifically, we aimed to gain an understanding of past and current SARS-CoV-2 infection rates in an asymptomatic workforce at Wright-Patterson Air Force Base (WPAFB) in Dayton, OH. The study consisted of two aims (Figure 1). Aim 1 focused on serological testing for past infection and included two sub-aims: investigating seroprevalence in the population (Aim 1a) and evaluating performance of multiple point-of-care (POC) lateral flow assays (LFAs) (Aim 1b). Aim 2 focused on molecular testing for active SARS-CoV-2 infection and evaluation of RT-LAMP as an alternative testing solution to RT-PCR. Participants were recruited from the WPAFB workforce and surrounding communities and had no known exposure or prior confirmed clinical COVID-19 diagnosis. Within this population, we determined a seroprevalance rate of 3.09% and an active infection rate of 0.5%, both of which were comparable to local and national averages at the time. Here, we also present technical and non-technical comparisons of the various testing strategies. This study was discussed during its early stages as part of a review delineating U.S. Air Force science and technology solutions for scalable SARS-CoV-2 testing (29).

## Materials and Methods

### Participant Enrollment

Study participants were recruited from the WPAFB workforce (military, civilians, and contractors) and surrounding communities using multiple media outlets. Participants had to be in healthy condition and asymptomatic with no known prior SARS-CoV-2 infection or exposure. Study participants were enrolled and consented by phone by a study investigator. To determine eligibility, participants provided answers to a secure COVID-19 screening and demographics questionnaire recorded in an electronic database (Smartabase; FusionSports, Boulder, CO). Participants were able to enroll in the serosurvey (Aim 1a or Aims 1a and 1b) and/or the molecular survey (Aim 2). Experiments were not completed in a Clinical Laboratory Improvement Amendments (CLIA) certified lab and participants understood the study would not provide diagnostic results and was solely research based. If participants were found to be at risk of COVID-19 due to exhibiting symptoms or being in close contact with a person diagnosed with COVID-19, or upon testing positive for SARS-CoV-2 infection in a saliva sample, participants were referred to their medical provider and excluded from the remainder of the study. This study was approved by the Air Force Research Laboratory Institutional Review Board (IRB) and conducted in accordance with the provisions of the Belmont Report, Common Rule, and Department of Defense Instruction 3216.02 Protection of Human Subjects guidelines. All study participants provided informed consent prior to enrollment.

### Serosurvey Sample Collection and Processing

#### Blood Sample Collection

Participants in Aim 1a could elect to collect their own samples at home using the MITRA® Home Blood Collection Kit developed by Neoteryx (Torrance, CA). This kit included a microsampling collection device, gauze, a lancet, all necessary shipping materials, and detailed instructions for participants to collect a blood sample. Briefly, each kit contained four swabs. After the finger-stick with the included lancet, ~20 μl of blood would be wicked into each of the four swabs for a total collected volume of 80 μl. Participants performed the blood collection at home and then shipped the sealed kit in the provided mailer to an off-site central collection site. The completed kits were collected weekly from the central location and brought to WPAFB for processing.

The remaining participants enrolled in Aim 1a, as well as participants enrolled in Aim 1b, provided venipuncture samples at a dedicated location ~2.3 miles away from WPAFB. Depending on sub-aim enrollment, one or two blood samples, with a combined volume of no more than 16 mL, were collected by a certified phlebotomist *via* venipuncture with a 21- or 23-gauge butterfly needle up to once every two weeks over an 8-week period. One sample was collected in a Serum Separation Tube (SST; 3.5 ml BD Vacutainer Venous Blood Collection Tubes: SST, BD 368015) for serum analysis by serological immunoassay and ELISA. For participants enrolled in Aim 1b, a second sample was collected in ethylenediaminetetraacetic acid (EDTA) blood tubes (3 ml BD Vacutainer Plastic Blood Collection Tubes: K_2_EDTA, BD 368,589) for whole blood analysis by LFA. Samples were transported to WPAFB in refrigerated containers within 2 h of collection.

#### Blood Sample Processing

The dried blood swabs (each containing ~20 μL of finger-stick blood) from the MITRA® Micro kits obtained *via* at-home collection were individually placed into wells of a Fisherbrand™ 96-Well DeepWell™ Polypropylene deep well plate containing 400 μl of 1% Bovine Serum Albumin (BSA), 0.5% Tween-20, and 1x phosphate-buffered saline (PBS). Plates were shaken using a digital microplate shaker at 300 rpm at 4°C overnight. Even though the level of Tween-20 (0.5%) was sufficient to inactivate any viruses, the samples were further heat-inactivated in a 56°C water bath for 45 min. Samples were aliquoted for storage at −80°C until enough samples were collected to run a full 96-well ELISA or immunoassay.

SSTs containing whole blood from on-site collection were centrifuged at 1000 × RCF for 10 min at room temperature (RT) using a Fisherbrand™ HORIZON™ 24 Flex Clinical Centrifuge designed for low RCF blood tubes in order to separate the serum from the whole blood. Serum was then aliquoted into pre-labeled 5 mL internally threaded cryo-tubes and heat-inactivated in a 56°C water bath for 45 min. Serum samples were aliquoted for storage at −80°C until assayed *via* ELISA or serological immunoassay. EDTA tubes were stored at RT and assayed *via* LFA the same day.

#### Lateral Flow Assays (LFAs)

LFAs from three different manufacturers were used to detect IgG and IgM in whole blood samples following each manufacturer's instructions. Tests included: (1) COVID-19 IgM/IgG Rapid Test from CareHealth America Corp., Blue Earth, MN (“CareHealth”), (2) Diagnostic Kit for Antibody IgM/IgG of Novel Coronavirus COVID-1 from AXON Connected, Earlysville, VA (“AXON”), and (3) Instant-view® IgG/IgM Antibody from Alfa Scientific Designs Inc., Poway, CA (“Alfa”). EDTA tubes containing whole blood were inverted to mix samples. A small amount of blood (10 to 20 μl per manufacturer's instructions) was pipetted directly onto the LFA device followed by 2–3 drops of running buffer supplied with the LFA kits. Devices were incubated at RT in a biosafety cabinet and results were read as per manufacturer protocols. Positive assays were repeated to confirm result.

#### Serological Immunoassay

Either 10 μL or 20 μL (as dictated by the individual manufacturer's assay protocol) of heat-inactivated serum samples (for IgM and IgG assays, respectively) were loaded onto a Beckman Coulter UniCel DxI 800 (Beckman Coulter Inc., Brea, CA) and evaluated according to manufacturer's instructions. The Beckman Coulter (BC) immunoassays are two-step immunocapture immunoassays that use chemiluminescence to detect a qualitative result in human serum or plasma. Daily maintenance and quality controls were conducted as per manufacturer's operating instructions, and each assay was calibrated every 28 days. For the IgM assay, results were interpreted based on Sample/Control SARS-CoV-2 IgM values as follows: Non-Reactive IgM (“negative”) if <1.00 or Reactive (“positive”) if ≥1.00. For the IgG assay, results were interpreted based on Sample/Control SARS-CoV-2 IgG values as follows: Non-Reactive result (“negative”) if ≤ 0.80, Equivocal (“gray zone”) if >0.80 and<1.00, or Reactive (“positive”) result if ≥1.00.

#### Enzyme-Linked Immunosorbent Assay (ELISA)

ELISAs were performed using a two-step method developed by Stadlbauer et al. (11). The method was modified by using 1x 3,3,5,5—tetramethylbenzidine (TMB) substrate and a weighted-ELISA analysis (described in detail below). The first phase of indirect-ELISA was performed using Klumpp-Thomas methodology (12). For each assay, three ELISA plates were run, each with a different secondary detection antibody (IgA, IgG, or IgM). A plasmid expressing the receptor binding domain (RBD) of the spike glycoprotein was produced under HHSN272201400008C and obtained through BEI Resources, NIAID, NIH: Vector pCAGGS Containing the SARS-Related Coronavirus 2, Wuhan-Hu-1 Spike Glycoprotein Gene RBD with C-Terminal Hexa-Histidine Tag, NR-52309. The plasmid was amplified in *Escherichia coli* in-house and sent to Fisher Scientific for transfection into mammalian cells, final protein purification, and validation. 50 μL of purified RBD [2 μg/mL in 1x PBS (Thermo Fisher, #AM9625)] was coated on each ELISA plate (Nunc MaxiSorp™ high protein-binding capacity 96 well ELISA plates, Thermo Fisher Scientific) and incubated at 4°C for a minimum of 16 h. ELISA plates were washed three times with 0.05% Tween-20 (Fisher Scientific, #J20605AP) in 1x PBS (PBS-T) then blocked with 5% non-fat skim milk in PBS-T (200 μL/well) for 2 h at RT. After incubation, blocking buffer was removed and 100 μL of 1:400 dilution heat-inactivated serum samples (diluted in 5% nonfat skim milk in PBS-T) were added per well in duplicate on each plate and allowed to bind for 1 h at RT. Plates were washed three times with PBS-T in an automated plate washer. 50 μL of detection antibody solutions (goat anti-human-IgA, IgG, or IgM from Thermo Fisher Scientific; 1:4000 dilution in PBS-T with 1% non-fat skim milk) were added to each well of their respective plates, and the plates were incubated for 1 h at RT. Plates were washed three times with PBS-T. 100 μL of TMB (Thermo Fisher Scientific, #34029) was added to each well to develop the assay for 10 min at RT, then 100 μL of 1N sulfuric acid stop solution (Thermo Fisher Scientific, #SS04) was added to stop the reaction. Optical density at 450 nm (OD_450_) was measured on either a FlexStation or BioTech spectrophotometer within 5 min of stopping the reaction. A sample was considered to be “presumptive positive” if the OD_450_ was higher than the mean OD_450_ plus 3 times the standard deviation (Mean OD_450_ + 3σ) of four negative serum samples for each ELISA plate. All ELISAs included SARS-CoV-2 negative serum as negative controls and deidentified convalescent plasma with known antibody-titer levels from verified COVID-19 patients, provided by Armed Services Blood Bank Center (Bethesda, Maryland), as positive controls.

Any presumptive positive samples were subjected to a second indirect ELISA using the antibody isotype that was serologically reactive from the first ELISA assay. The second ELISA was performed using a similar method modified from Stadlbauer et al. (11). For each assay, ELISA plates were run using a different secondary detection antibody (IgA, IgG, or IgM). A plasmid expressing the full spike glycoprotein was produced under HHSN272201400008C and obtained through BEI Resources, NIAID, NIH: Vector pCAGGS Containing the SARS-Related Coronavirus 2, Wuhan-Hu-1 Spike Glycoprotein Gene (soluble, stabilized), NR-52394. As with the RBD, the plasmid was amplified in *E. coli* in-house and sent to Fisher Scientific for transfection and purification. 100 μL of purified spike protein (1 μg/mL in 1x PBS) was coated on each ELISA plate and incubated at 4°C for a minimum of 16 h. ELISA plates were washed three times with PBS-T then blocked with 3% non-fat skim milk in PBS-T (200 μL/well) for 1 h at RT. After incubation, blocking buffer was removed. Heat-inactivated serum samples were diluted 1:5 in 1x PBS then 1:100 in 1% non-fat skim milk in PBS-T. Serum samples were then serially diluted (four additional 3-fold dilutions) within the ELISA plate and then incubated for 2 h at RT. Plates were washed once with PBS-T. 50 μL of detection antibody solutions (goat anti-human-IgA, IgG, or IgM from Thermo Fisher Scientific; empirically-derived dilution of 1:10000 in PBS-T with 1% non-fat skim milk) were added to each well of their respective plates, and the plates were incubated for 1 h at RT. Plates were developed and measured as described above.

Any samples that had at least two OD_450_ values greater than the cut off OD_450_ within the same serial dilution series were considered to be positive for that particular antibody. The cut-off OD_450_ value was calculated as the mean OD_450_ plus 3 times the standard deviation (Mean OD_450_ + 3σ) of serially diluted negative pooled serum samples on the same plate. To limit false positives, we established a weighted-ELISA (W-ELISA) approach. To do so, control samples of convalescent plasma from confirmed COVID-19 patients were evaluated by the two-step ELISA. The convalescent plasma samples consistently crossed the cut-off OD_450_ value for three or more serial dilutions on the second ELISA. Using that information, we determined that a sample needed to cross the cut-off OD_450_ in three of five serial dilutions to be considered positive. The W-ELISA was considered the standard by which the BC immunoassays and LFAs were evaluated against.

### Saliva Sample Collection and Processing

#### Saliva Sample Collection

Participants were instructed to refrain from eating, drinking, chewing gum, and using tobacco for 30 min prior to sample collection. Participants self-collected their samples using a DNA/RNA Shield Saliva Collection Kit (Zymo Research, Irvine, CA). Briefly, participants deposited ~2 mL saliva into a tube containing a 2 mL solution which inactivated the virus and preserved the viral nucleic acid. Samples were collected at a dedicated location ~2.3 miles from WPAFB and transported to WPAFB within 2 h of collection.

#### Saliva Sample Processing

Analysis of 704 samples processed individually over the first four weeks of sampling uncovered no positive samples. Based on the demonstrated low prevalence of active infection, sample pooling of 5 samples per pool was adopted to more efficiently use testing supplies during the remainder of the study. Samples were pooled by pipetting 200 μL of up to five samples into a 2 mL cryotube. Proteinase K (PK; Thermo Fisher) was added to each tube at a 1:200 dilution (e.g., 5 μL PK to 1 mL saliva) and tubes were mixed by inversion and incubated at 65°C in an oven (Thermo Scientific Heratherm™) for 90 min to further heat inactivate the samples and aid in pipetting. Samples were immediately used for RNA extraction. Samples unable to be assayed on the day of receipt were stored at RT until they could be processed, typically for <24 h. In the event of a positive pool, processing and subsequent steps were repeated using unpooled, individual samples.

#### RNA Extraction

RNA extraction was automated on a KingFisher™ Flex (Thermo Fisher) using the MagMAX™ Viral/Pathogen II Nucleic Acid Isolation Kit (Thermo Fisher) run following manufacturer's protocol and directions in the associated Emergency Use Authorization (EUA) (30) using KingFisher™ Deepwell 96 Plates set up as follows. The first wash plate contained 500 μL/well of MagMAX™ Viral/Pathogen Wash Solution. The second wash plate contained 1 mL/well of freshly prepared 80% ethanol. The elution plate contained 50 μL/well of MagMAX™ Elution Solution. Magnetic bead solution was made fresh daily by mixing Total Nucleic Acid Magnetic Beads with Binding Solution at a ratio of 10 μL:265 μL (mixed by gentle inversion to avoid bubbles). To prepare the sample plate, the following was added in order: 5 μL of MS2 Phage Extraction Control, 275 μL magnetic bead solution, and 200 μL processed saliva sample. For a negative control, 200 μL water was added in place of sample. All plates were loaded on the KingFisher™ Flex along with a KingFisher™ 96 Tip Comb (Thermo Fisher) and run through the MVP_2Wash_200_Flex protocol (30). Eluted RNA was kept on ice until assayed using RT-PCR and RT-LAMP.

#### Reverse Transcription Polymerase Chain Reaction (RT-PCR)

RT-PCR reactions were performed using the TaqPath™ RT-PCR COVID-19 Kit (Thermo Fisher), which targets three SARS-CoV-2 gene targets (N gene, S gene, and ORF1ab) and a MS2 phage extraction control. Master mix was prepared in the following ratios per reaction: 5 μL of TaqPath™ 1-Step Multiplex Master Mix (No ROX™) (4X), 1 μL COVID-19 Real Time PCR Assay Multiplex, and 4 μL Nuclease-free Water. 10 μL of reaction master mix was added to each well in a 96-well MicroAmp™ Fast Optical 96-well Reaction Plate followed by 10 μL of eluted RNA. Eluted negative control was added to the negative control well. Positive Control (synthetic standard included in kit) was freshly diluted per EUA instructions with Positive Control Dilution Buffer, and 10 μL was added to the positive control well. The plate was sealed with MicroAmp™ Optical Adhesive Film, mixed at 1,750 rpm on a Q-Instruments Bioshake IQ for 3 s, and centrifuged for 1 min. The RT-PCR reaction was run on a QuantStudio™ 7 Flex Real-Time PCR Instrument using cycling conditions from the EUA: 25°C for 2 min, 53°C for 10 min, 95°C for 2 min, 40 cycles of 95°C for 3 s and 60°C for 30 s. Results were analyzed using Design and Analysis Software (Thermo Fisher, Ver 2.4) using Presence/Absence analysis with interpretative rules provided by the manufacturer under the EUA. Viral gene targets were considered present if the Ct value was ≤ 37. Briefly, if two or more SARS-CoV-2 gene targets were detected, the sample/pool was called as positive. If MS2 was detected but no SARS-CoV-2 gene targets were detected, the sample/pool was called as negative. If all gene targets, including the MS2 phage, were undetected in a reaction, it was considered invalid and the sample/pool was reextracted and reassayed. If only one SARS-CoV-2 gene target was detected, the reaction was considered inconclusive and repeated. For pooled samples, if a pool was positive or inconclusive, RNA was extracted from the individual samples and assayed in triplicate using both RT-PCR and RT-LAMP. In the event of a positive result, the subject was alerted by the IRB-assigned medical monitor and referred to their healthcare provider for additional guidance and subsequently removed from the study.

#### Reverse Transcription Loop-Mediated Isothermal Amplification (RT-LAMP)

A solution was prepared by mixing: 8.75 μL 100 mM dUTP, 25 μL 1000 U/mL UDG, 1.25 μL SYTO-9, and 1.25 mL WarmStart Colorimetric LAMP 2X Master Mix (New England Biolabs). A subsequent master mix was prepared in the following ratios per reaction: 10 μL of the initial solution, 2 μL LAMP primer mix (10X) [containing 6 Gene N-B primers from (25)], and 0.1 μL 8 M guanidine hydrochloride. 12 μL of the master mix was added to each well of a 96-well MicroAmp™ Fast Optical 96-well Reaction Plate followed by 10 μL of extracted RNA, including eluted negative control. For a positive control, 1000 copies of Synthetic SARS-CoV-2 RNA (Twist Biosciences, 102019) was added to the positive control well. The plate was incubated at 25°C for 2 min to eliminate contamination from previous runs then at 65°C for 30 min. Fluorescence was measured in real time and a subsequent melt curve analysis was performed on a QuantStudio™ 7 Flex Real-Time PCR Instrument. Reactions were considered positive if the fluorescence crossed threshold.

#### Assay Characterization and Verification

To characterize the RT-PCR assay in saliva, contrived samples were generated by diluting AccuPlex™ SARS-CoV-2 Full Genome Control (SeraCare, 0505-0159) into saliva known to be negative for SARS-CoV-2. All standard samples were extracted and assayed according to manufacturer instructions. For comparison of RT-PCR and RT-LAMP assays, dilutions of Synthetic SARS-CoV-2 RNA Control (Twist Biosciences, 102019) in water were extracted and assayed. Synthetic standards of known concentration were used for quality control of the RT-PCR assay throughout the study as well.

### Statistical Analysis

To compare serological test methods, data was analyzed using a Repeated Measures ANOVA with a *post hoc* Dunnett's test compared to the W-ELISA data (GraphPad Prism 9.0.2).

## Results

### Participant Characteristics

Samples were analyzed from 654 asymptomatic adults working at WPAFB or the surrounding area. Of the total participants, 582 took part in Aim 1. 566 of these participants provided questionnaire data (Table 1), and 1,568 blood and/or serum samples were analyzed for the presence of SARS-CoV-2 antibodies. Of the total participants, 404 took part in Aim 2. 342 of these participants provided questionnaire data, and 3,236 saliva samples were analyzed for SARS-CoV-2 viral RNA.

**Table 1:** Population characteristics for participants who provided questionnaire responses.

	**Study Population**	
	***n***	**%**
**Sex**		
Male	366	62.03
Female	224	37.97
**Race**		
White only	515	87.59
Black only	16	2.72
Others	57	9.69
**Age group**		
18–44	313	48.15
45–69	313	48.15
70–95	24	3.69
**Employment**		
Employed	530	90.60
Unemployed	5	0.85
Student	14	2.39
Retired	32	5.47
Homemaker	4	0.68
**Homeowner**		
Own	460	78.36
Rent	114	19.42
Others	13	2.21
**Flu vaccinated**		
Yes	317	53.82
No	272	46.18
**Myocardial infarction**		
Yes	6	1.03
No	575	98.97
**Angina or heart disease**		
Yes	10	1.71
No	574	98.29
**Stroke**		
Yes	7	1.20
No	574	98.80
**Asthma**		
Yes	72	12.44
No	507	87.56
**Still have asthma**		
Yes	28	49.12
No	29	50.88
**Skin cancer**		
Yes	43	7.43
No	536	92.57
**Other cancer**		
Yes	24	4.12
No	559	95.88
**COPD/emphysema/chronic bronchitis**		
Yes	4	0.69
No	575	99.31
**Arthritis/Rheumatoid Arthritis/lupus/fibromyalgia**		
Yes	110	19.26
No	461	80.74
**Depression/dysthymia**		
Yes	79	13.67
No	499	86.33
**Kidney disease**		
Yes	3	0.51
No	581	99.49
**Diabetes**		
Yes	23	3.95
No	560	96.05
**Diabetes diagnosis age**		
18–45	8	34.78
45–70	15	65.22
70–96	0	0.00

### Aim 1: Serological Survey

#### Prevalence of Past Infection

**Figure 2:**
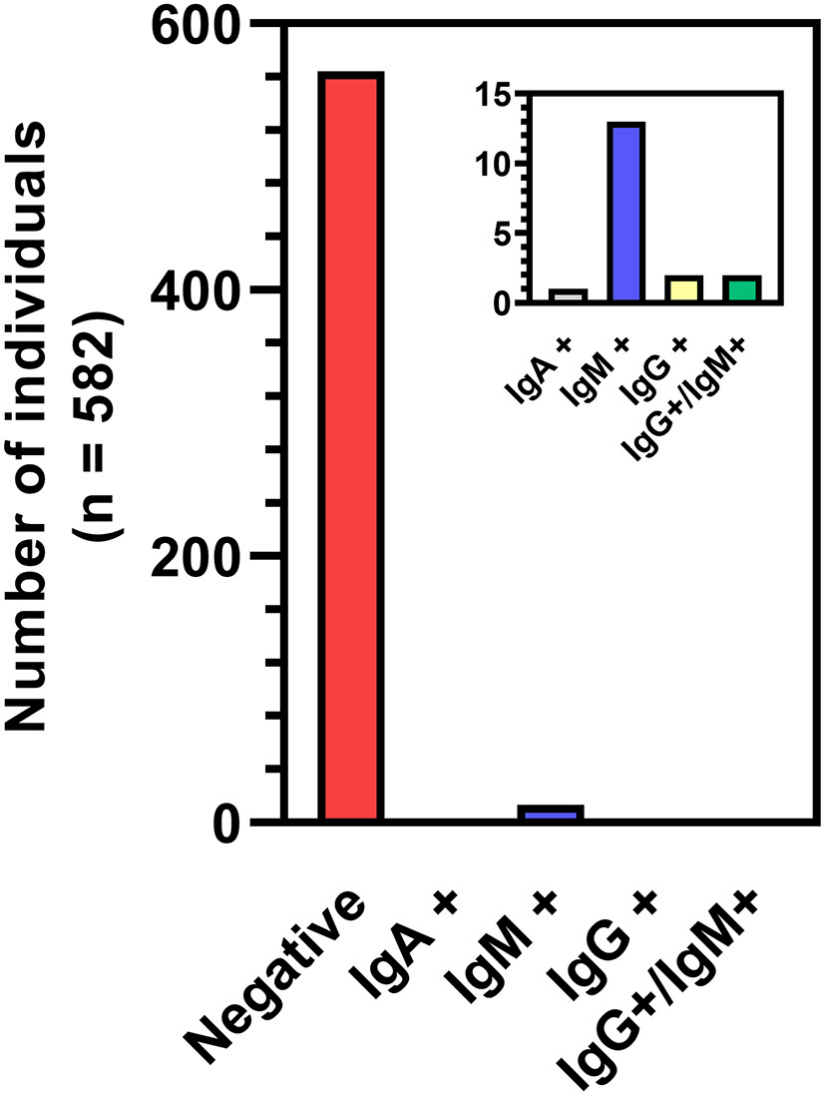
Breakdown of seropositive participants based on W-ELISA. The two participants represented by the IgG+/IgM+ bar had a sample test positive for both antibodies. All other positive participants tested positive to the same single antibody in one to four blood samples.

A total of 1,568 blood samples were analyzed from 582 participants over the span of three months (23 Jul 2020 to 23 Oct 2020), with each participant providing up to four samples spaced about every two weeks. Of the total samples, 27 were collected *via* self-administered finger-stick using a MITRA® blood collection kit and 1,541 were collected *via* venipuncture. All samples were analyzed using a two-step ELISA, testing first for binding to the receptor binding domain (RBD) of the SARS-CoV-2 spike protein then for binding to the full spike protein. All 27 finger-stick samples were negative on the first ELISA and were removed from subsequent analyses. 3.0% of remaining samples (46/1541) were determined to be IgG positive in the two-step ELISA, coming from 28 individuals. To reduce the probability of a TYPE II statistical error (i.e. a false positive), we used a “weighted” methodology to re-analyze the samples with a weighted-ELISA (W-ELISA) approach (see Methods). Using the W-ELISA approach, only 8 samples were IgG positive, coming from 4 participants (Figure 2). Furthermore, 1 sample coming from 1 participant and 20 samples coming from 15 participants were IgA or IgM positive, respectively. In most cases, samples were only positive for one antibody, with the exception of two samples that were positive for both IgG and IgM. In addition, most participants only had one or two positive samples throughout the study while two participants had all four samples test positive, either for IgG or IgM, respectively. Taken altogether, 3.09% of participants sampled (18/582) had one or more serologically reactive antibodies for the SARS-CoV-2 spike protein at some point during the study.

#### Serological Assay Comparison

**Figure 3:**
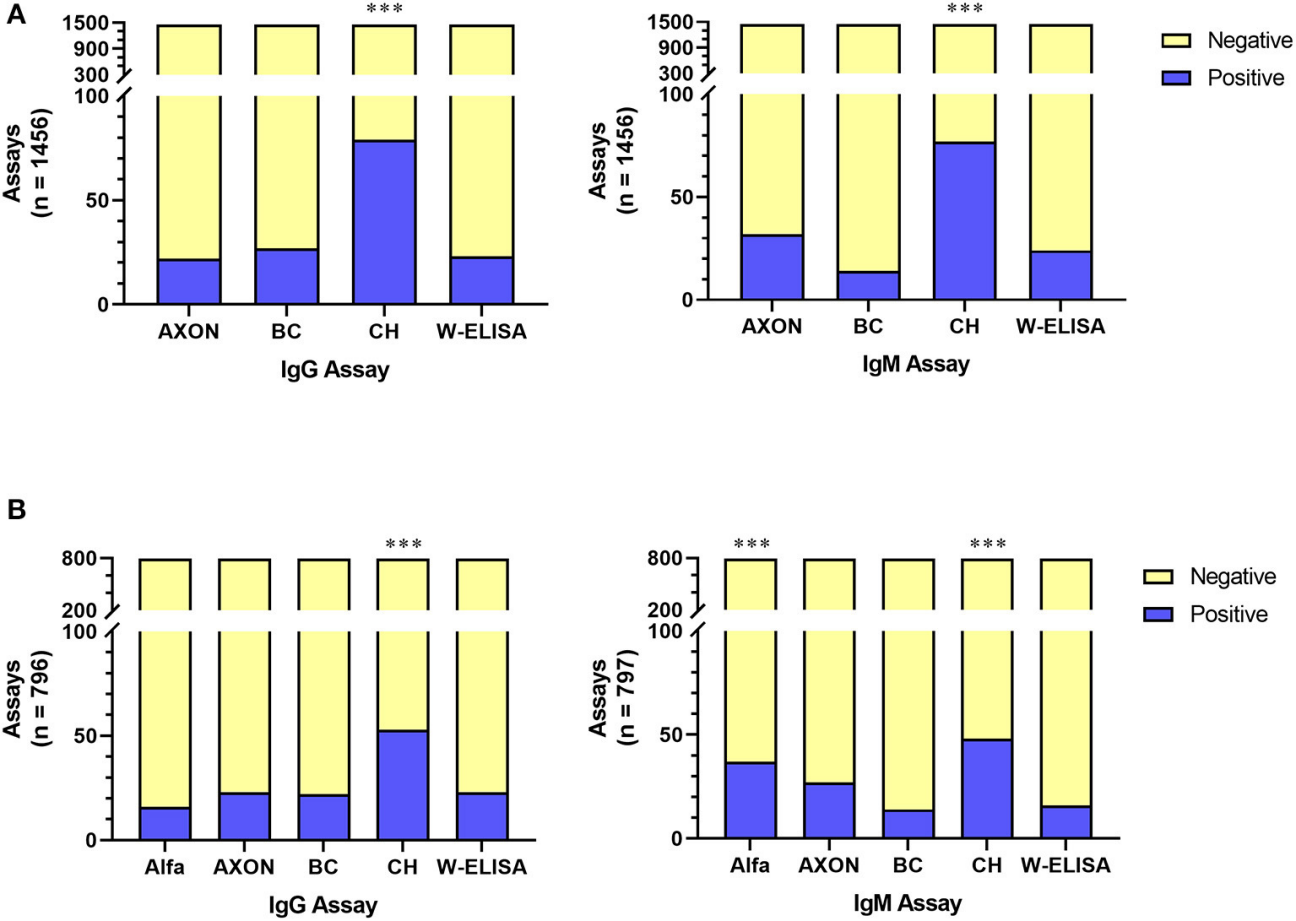
Comparison of serological tests for both IgG and IgM antibodies across either 4 assays **(A)** or 5 assays **(B)**. In all comparisons, 20 assays were conducted on control samples and the remainder was on participant samples. Test performance was compared to the W-ELISA data; *p* < 0.001 (^***^). Alfa, Alfa LFA; AXON, AXON LFA; BC, Beckman Coulter immunoassay; CH, CareHealth LFA; W-ELISA, Weighted ELISA.

To independently validate various antibody testing assays, 1,436 whole blood samples were tested using four assays: W-ELISA, POC LFAs from AXON and CareHealth, and a Beckman Coulter (BC) immunoassay. All assays were compared to the gold standard W-ELISA reference using a Repeated Measures ANOVA in order to compare the W-ELISA result with the corresponding POC/BC assay result for the same sample (Figure 3A). For both IgG and IgM evaluation, the AXON LFA and the BC immunoassay performed statistically similar to the W-ELISA, whereas the CareHealth LFA was significantly different. For ~787 of the samples, an additional LFA from Alfa was also evaluated. When comparing the evaluation of all five assays within these samples, for IgG the only assay that showed significant difference was the CareHealth LFA, while the rest performed similarly. However, for IgM evaluation, the CareHealth and Alfa LFAs both performed differently than the W-ELISA, while the BC assay and AXON LFA performed similarly (Figure 3B). In summary, the AXON LFA and BC assay were the only tests to reliably produce statistically similar results as the W-ELISA standard.

### Aim 2: Molecular Testing Using Saliva

#### RT-PCR Assay Characterization

**Figure 4:**
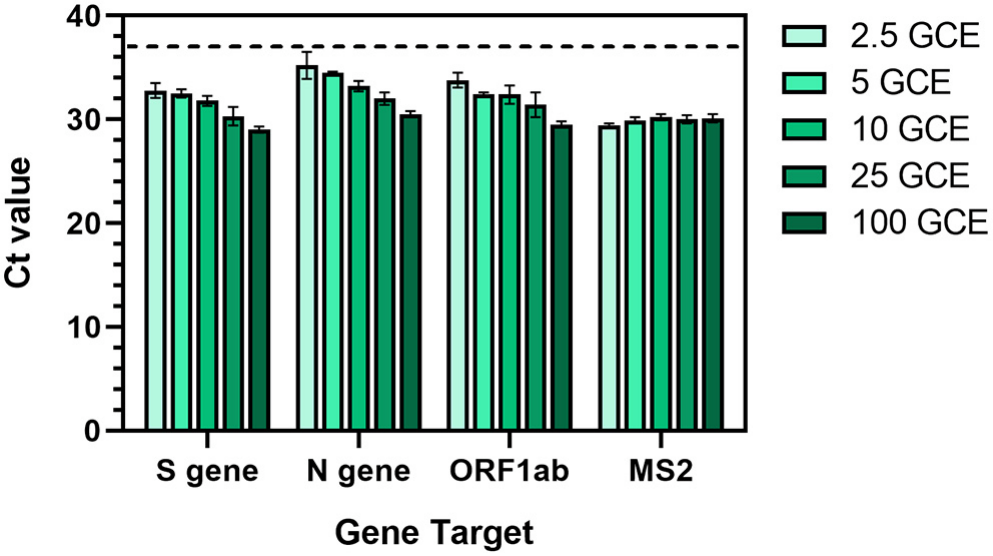
Characterization of Thermo Fisher TaqPath™ RT-PCR COVID-19 assay using contrived positive saliva samples with a range of SARS-CoV-2 genomic copy equivalents (GCE). An equal amount of MS2 phage was added to each sample as an extraction control. Values represent Ct values ± standard deviation of three reactions. Dotted line represents the detection threshold (Ct = 37).

Thermo Fisher's TaqPath™ COVID-19 Combo (“TaqPath”) Kit has been adopted in many COVID-19 testing EUA protocols (31). Specifically, the assay tests for the presence of three SARS-CoV-2 genes (N gene, S gene, and ORF1ab) in multiplex along with an internal extraction control (MS2 phage). The claimed limit of detection for the TaqPath kit is 10 genomic copy equivalents (GCE) per reaction (30), although actual performance has been shown to vary (32). To characterize the assay in-house, we contrived artificial positive samples by diluting standards into saliva known to be negative for SARS-CoV-2. The original claims in the EUA to detect virus down to 10 GCE per reaction were confirmed (Figure 4), with the assay detecting gene targets down to 2.5 GCE per reaction. The assay performed reliably within 10–25 GCE in quality control tests throughout the study using various known standards.

#### Comparing Testing Methods for Active Infection

**Figure 5:**
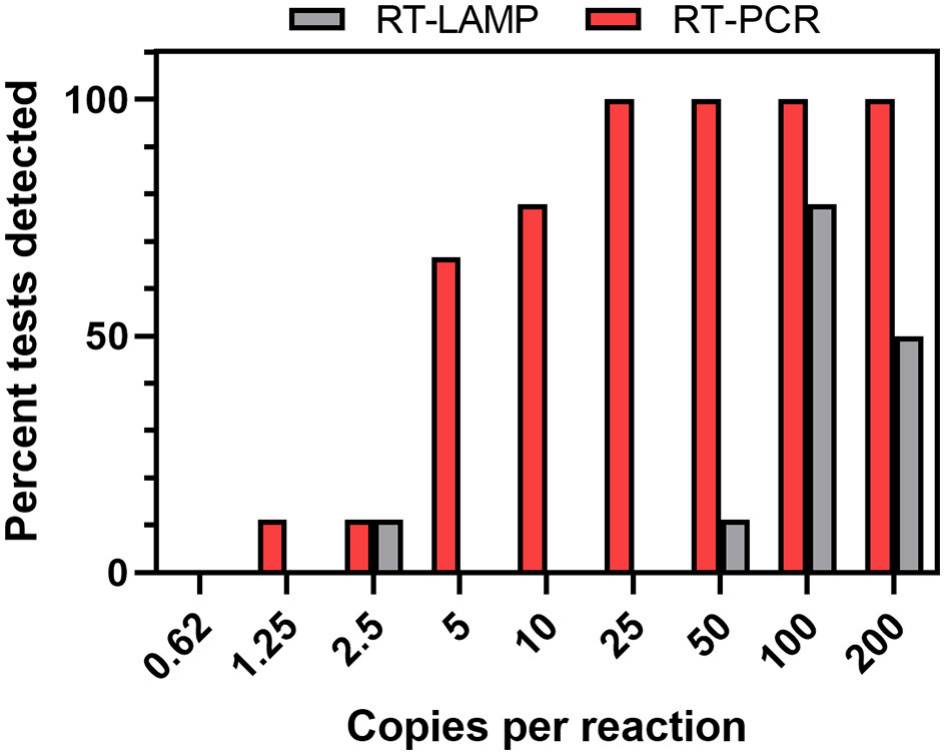
RT-PCR and RT-LAMP comparison using synthetic standards. Dilutions of synthetic control RNA in water were extracted and assayed (*n* = 4 or 9 tests per dilution). For RT-PCR assays, the viral genes were considered detected if 2 of the 3 viral genes were detected.

RT-LAMP was evaluated as an alternative assay to RT-PCR. We initially utilized RT-LAMP conditions using a commercial mix and various published LAMP primer sets testing a dilution series of Synthetic SARS-CoV-2 RNA. In this study, the Gene N-B primer set (25) performed well without the frequent presence of false positives. We optimized the assay further through the additions of: (1) double-stranded DNA fluorescent dye SYTO-9 to directly detect amplification instead of relying on the colorimetric pH indicator provided in the mix (33), (2) guanidine hydrochloride to increase sensitivity (34), and (3) deoxyuridine triphosphates and uracil DNA glycosylase (dUTP/UDG) to reduce carryover contamination between runs (35). We compared the two assays using synthetic SARS-CoV-2 RNA dilutions ranging between 0.625 and 200 copies per reaction (Figure 5). RT-PCR outperformed RT-LAMP in sensitivity of detection, with RT-LAMP only detecting the gene target in ~70% of the reactions with ≥100 copies per reaction, drastically dropping in performance at <100 copies. Notably, the TaqPath assay being multiplexed detects 3 SARS-CoV-2 genes, while the RT-LAMP assay only detects the N gene. In this analysis, a reaction was considered detected by RT-PCR if two of the three genes were detected.

#### Prevalence of Active Infection

A total of 3,236 saliva samples were collected from 404 participants over the span of ~9 weeks (18 Aug 2020 to 23 Oct 2020) with each participant providing up to 16 samples spaced about twice weekly. Active infection was evaluated by testing for the presence of SARS-CoV-2 genes using RT-PCR assay and RT-LAMP. Using RT-PCR, only 2 participants emerged as positive over the span of the study (Table 2), while the rest were negative throughout. The positive samples were originally detected from pools of 5 and then identified by reassaying individual samples from these pools, suggesting that the assay was sensitive enough with the pooled approach, even with a low titer sample (Sample B). Of note, the initial pooled RT-PCR reaction only detected the ORF1ab gene target in Sample B, while reassays on the individual sample detected 2 or more gene targets in triplicate reactions, showcasing the utility of multiplexed reactions for viral gene detection. All three genes were detected in both the pool and individual reassay for Sample 1. Additionally, RT-LAMP only detected the presence of viral RNA for the higher titer sample, but failed to detect viral RNA in the low titer sample both in the pool and individually, suggesting that participants with a low level of infection may be missed by RT-LAMP. Both positive pools only contained one positive sample.

**Table 2:** Positive SARS-CoV-2 samples and associated Ct values from the RT-PCR assay.

	**S gene**	**N gene**	**ORF1ab**	**MS2**
Positive sample A	26.8	27.5	26.1	30.4
	27.2	27.9	26.7	30.7
	27.1	27.8	26.6	30.9
Positive sample B	34.2	nd	33.7	32.5
	35.3	33.7	36.1	32.3
	nd	33.9	34.4	31.7

*Individual samples from positive or inconclusive pools were reassayed in triplicate, reported here. nd = not detected*.

## Discussion

The purpose of this study was to demonstrate evidence-based solutions for advanced, widespread, rapidly available testing in a large workforce population in order to inform return to full capacity decisions in the face of the COVID-19 pandemic. Using the gold standard approaches, we were able to determine past exposure and current infection levels in an asymptomatic population. In addition, we investigated multiple alternative approaches for both serological and molecular testing, comparing them to the current standards.

### Aim 1: Serological Testing

Knowledge of seroprevalance levels in a population can be used to formulate responses to current and future pandemics. However, reporting an erroneous positive has far-reaching implications. For example, if an individual assumes they have reactive antibodies (based on a false positive), they may assume they have some prophylactic immunity against SARS-CoV-2. The false assumption of immunity may translate into higher risk-based behaviors. Therefore, in order to prevent false positives in our assay, we used an antibody-titer approach to determine cut-off conditions for our W-ELISA. This afforded us high confidence in the seropositivity rates obtained in the study. It's also important to note that we only evaluated for the presence and isotype of antibodies, and future studies are required to infer immunity.

Using our W-ELISA approach, we observed a 3.09% seropositivity rate in unexposed members of the WPAFB community. The National Institutes of Health (NIH) conducted a similar, large scale study shortly before the timeframe as this study (10 May 2020–31 July 2020 and 23 Jul 2020–23 Oct 2020, respectively) (6). While they found the average seropositivity rate in undiagnosed adults in the Midwest to be 1.6% (95% CI: 0.3–2.4), the national average was 4.6% (95% CI: 2.6–6.5%). Notably, the Midwest had a lower new case rate during the span of the NIH study, with cases ticking up in July 2020, plateauing for the most part for the majority of the present study, before significantly increasing starting in mid-October leading into the winter surge. Therefore, the levels of seroprevalance seen in this study appear to be typical of the national average at the time. Other factors potentially affecting the seropositivity rate include the fact that WPAFB and many businesses in the area were encouraging telework during the span of the study, the fact that participation criteria excluded those that had a known previous infection, and the fact that the cohort only included asymptomatic individuals.

#### Point of Care Antibody Testing Assay Evaluation

Of note, in this study most positive individuals had reactive antibodies for IgM or IgG, with the majority being IgM-positive. While IgM presence can signal recent exposure/infection, in this study most participants who had IgM-positive sample(s) had subsequent negative sample(s). In the event of a true infection, one would expect subsequent samples to be IgM and/or IgG positive. The transient expression of IgM without conversion to IgG could either reflect a low-level exposure neutralized by IgM alone or the known cross-reactivity of IgM. The authors caution the reader that IgM is well known to be a problematic capture and/or detection antibody due to the inherent nature of the immunologic function of the IgM isotype. The use of IgM as a detection antibody is well associated with false positives primarily due to the cross reactivity of IgM (36, 37). Research to decrease IgM-related false positives in ELISA-based assays specific to SARS-CoV-2 is ongoing (38).

While ELISAs are a reliable and sensitive standard for antibody detection, a laboratory-based method is not always practical in operational settings. Therefore, we collaborated with the Air Force Life Cycle Management Center (AF LCMC), the Joint Program Executive Office for Chemical, Biological, Radiological and Nuclear Defense (JPEO-CBRND), and the Naval Health Research Center (NHRC) to evaluate the performance of three different U.S.-manufactured POC LFA kits (CareHealth, AXON, and Alfa) to identify the optimal POC device for use in an operation field environment that requires minimal technical skills to use and evaluate. In concert with the POC evaluations, we evaluated the effectiveness of the immunoassay run on the Beckman Coulter UniCel DxI 800 (BC) chemical analyzer. The BC analyzer can accommodate up to 400 samples per hour, is EUA approved, and requires minimal staffing. However, reliable POC tests, such as LFAs, are desirable for widespread testing. Although sensitivity in LFAs is lower than clinical testing, especially in early onset of infection, these devices are less expensive, allow for cheap mass production, are easy to use at home or in the field, and provide rapid results in as little as 15 min (39). The manufacturer-claimed sensitivity and specificity, respectively, for the LFAs tested here are 90 and 100% (AXON), 93.5 and 100% (CareHealth), and 97.8 and 94.6% (Alfa), compared to the 99 and 99% seen in ELISAs (12). In this study, we independently evaluated the performance of the LFAs and BC immunoassays, compared to the reference W-ELISA. Assays were evaluated qualitatively against the gold standard W-ELISA to aid in determining usefulness for informing return to full capacity planning. For both IgG and IgM evaluation, the only assays to reliably produce statistically similar results as the W-ELISA standard were the AXON LFA and BC immunoassays. In terms of ease of use, when compared to the other evaluated LFAs, the AXON POC test required the lowest sample volumes, used the lowest diluent buffer volume, and had readable results within 10–15 min. Therefore, of the POC assays tested here, the AXON LFA appeared to be the most suitable for practical use in the operational setting.

In this study, we used whole blood to evaluate the LFA devices. The manufacturer's instructions called for the use of finger-stick blood; however, serum or whole blood could also be used. We verified with the manufacturers that EDTA (an anti-coagulant) in a blood collection tube would not hinder the assay. Whole blood was selected as the test medium for the LFA devices as whole blood had the greatest probability of confounding the assays due to the presence of red blood cells. Serum was used in the BC immunoassays, as per the manufacturer's protocol, as well as in the ELISAs as per previously established protocols. While the SARS-CoV-2 antibody response has been most well-studied in blood and serum, there's also been interest in using other biofluids, such as saliva. Using saliva for antibody detection has many advantages over blood/serum including requiring significantly less invasive sample collection and requiring fewer highly-trained personnel, making it a more easily fieldable option. While more studies need to be conducted, early studies show promising correlations between SARS-CoV-2 antibodies in serum and saliva (40, 41).

### Aim 2: Molecular Testing

In the second aim of this study, RT-PCR was performed on saliva samples utilizing a protocol derived from an EUA for Thermo Fisher's TaqPath™ COVID-19 Combo Kit (30). The TaqPath kit was among the earliest multiplex RT-PCR-based nucleic acid tests approved for detection of active SARS-CoV-2 virus (42), and it has since been widely adopted in clinical testing (31). Here, we independently characterized the TaqPath assay and used it to identify the prevalence of active SARS-CoV-2 infection in asymptomatic individuals using saliva samples as an alternative to the standard NP swab. Saliva samples were evaluated from a cohort of 404 self-reported asymptomatic individuals working at WPAFB and the surrounding area. Of these individuals, only 2 presented as positive over the duration of the study. The two positive samples were identified in pools of 5 samples then confirmed as individual samples in triplicate, demonstrating the sensitivity of RT-PCR to low viral titers. The TaqPath assay used has a calculated sensitivity of 97.8% (43) however, no false positive or false negative results were reported in this study. Taken together, the results from this aim demonstrate: (1) the utility of saliva as an analytical matrix for testing for SARS-CoV-2, (2) the value of pooling for resource and cost efficiencies, and (3) the value of a multiplexed assay that demonstrated the ability to detect an extremely low viral titer in the context of a pooled set of samples.

The frequency of active infection over the span of the study (~0.5%; 2/404) was similar to what was present statewide in Ohio in asymptomatic populations (0.9%; 95% CI: 0.1–2.0%) shortly before the study (18 Aug 2020–23 Oct 2020 compared to July 2020) (44). As noted above, cases in Ohio started increasing in July 2020, plateauing for the most part for the majority of this study, before significantly increasing starting in mid-October, as this study was winding down. The rate of active infection seen here at a time when telework was maximally encouraged, along with the fact that ~3.09% of participants had reactive antibodies, highlights the fact that asymptomatic and/or pre-symptomatic infection is a significant concern, and surveillance monitoring is incredibly important for returning to full capacity, especially in work environments such as the military, where there are high levels of interaction within the workforce.

Frequent testing and surveillance monitoring of infection is a vital tool for real-time monitoring of infection spread and prevention of outbreaks. This is exemplified by the results of this study. Here, participants were tested for active infection up to twice a week for 8 weeks. The two positive samples were the fifth or seventh sample collected from their respective participants, having had negative samples until then. This fact, added to the low viral titer of the second positive sample, shows how strong pooled surveillance testing can be in catching early infections in the workplace. In addition, the study protocol required participants to withdraw upon receiving a positive test as part of the study or as a result of a clinical test elsewhere. Over the course of the study, there were no withdrawals from participants who tested negative during the study supporting the idea that both the frequency of testing and analytical approach was effective in discerning their absence of virus.

Here, we used saliva samples for molecular testing for active SARS-CoV-2 testing. While NP swabs were the preferred method of sample collection early in the pandemic, other samples including oropharyngeal (throat) swabs, anterior nasal swabs, nasopharyngeal/nasal aspirate or wash, saliva, and even lower respiratory tract samples have become acceptable by the U.S. Centers for Disease Control and Prevention (45). Most of these sample collection methods require trained healthcare personnel and/or numerous supplies, including viral transport media for preservation. Saliva samples require none of these things, presenting the most promising option for widespread population surveillance testing. In fact, many universities and communities successfully used saliva for mass-scale surveillance testing throughout the pandemic.

#### Comparison of RT-LAMP and RT-PCR

We also evaluated RT-LAMP as an alternative protocol to RT-PCR. When testing assay sensitivity using synthetic SARS-CoV-2 samples, RT-PCR outperformed RT-LAMP in sensitivity of detection, and in the two positive saliva samples RT-LAMP only detected the viral gene target in the sample with the higher titer. This suggests that participants with a low level of infection may be missed by RT-LAMP. However, RT-LAMP is cheaper, easier to run, can produce positive results in shorter turnaround time, and requires less-sophisticated laboratory equipment, making it attractive for use in harsh operational environments. Conventional RT-LAMP has many limitations as well, as it is: (1) not quantitative, and is thus unable to provide insight into viral titer levels, (2) is difficult to multiplex, and (3) can be highly sensitive to the sample matrix (e.g., sample pH) resulting in false positives. Researchers have made great strides in protocol developments to remove these limitations however as well as simplifying the method by removing the need for RNA extraction or optimizing the method for saliva testing (46–53).

Another factor that affects testing choice is cost. In this study, we utilized saliva collection tubes from Zymo Research containing viral preservative solution. In addition to being susceptible to supply chain shortages, another drawback to using these or similar devices is price, as the price of the collection tube is on par with the assay costs. However, new techniques, such as those pioneered by Yale University and the University of Illinois (54–56), substantially reduce collection and processing costs by using widely available 50 mL conical tubes for collection as well as removing the RNA extraction step altogether (Table 3). Other cost-saving measures can involve aspects of the detection assay itself, through using RT-LAMP vs. RT-PCR, as well as using non-multiplexed primer sets. While widespread RT-PCR testing protocols tend to use primers targeting the single N gene, even within the small number of positive samples detected in this study, we saw the utility of multiplexing. Specifically, the second positive pool only resulted in detection of the ORF1ab gene target. Upon reassaying the individual sample in triplicate, 2 or more gene targets were detected in each reaction. Finally, no matter the protocol details, pooling samples drastically reduces costs, decreases supply chain limitations, and increases throughput, especially in cases of low active infection prevalence (57). In fact, pooled testing is becoming the go-to surveillance approach at this point in the pandemic (58, 59). In conclusion, as each assay and protocol has advantages and disadvantages, the decision of testing strategy usage will highly rely on specific circumstances, resources, and sensitivity needs.

**Table 3:** Cost per sample comparison across different collection and processing methods.

	**Testing individual samples**	**Testing in pools of 5**
Collection tube		
Zymo DNA/RNA shield	$12.50	$12.50
50 ml conical tube	$1.00	$1.00
RNA extraction cost	$2.96	$0.59
Assay cost		
RT-LAMP	$2.60	$0.52
MultiPlex RT-PCR	$13.28	$2.66
Total cost		
Zymo, extraction, RT-PCR	$28.74	$15.75
Zymo, extraction, RT-LAMP	$18.06	$13.61
50 mL, extraction, RT-PCR	$17.24	$4.25
50 mL, extraction, RT-LAMP	$6.56	$2.11
50 mL, RT-PCR	$14.55	$3.66

For various reasons, including those outlined above, RT-PCR has been the method of choice for widespread COVID testing to date, accounting for >75% of nucleic acid tests granted EUAs by the U.S. Food and Drug Administration (15). In this work, we demonstrated the ability to process several hundred samples at a time and return a result in ~6–8 h. A substantial amount of the time involved sample ingestion and reformatting from a low throughput format of a tube to a high throughput format of a 96 well plate. Once in a 96 well format, automated RNA extraction required ~23 min and the RT-PCR assay required ~1 h. To increase throughput further, a 384 well format can be used, and with pooling 1,920 individual samples could then be tested with results reported every hour. In a 24 h period with a single RT-PCR machine, this would suggest 9,168 individual samples could be tested individually, or 45,840 samples when pooled in sets of five; however, this requires highly efficient processing of samples. Automation of the upstream sample processing bottleneck has the potential to move this from theoretical possibility to practical reality. In fact, Thermo Fisher recently received an EUA to use a highly automated process (requiring 4 people per shift) to process up to 8,000 samples per 24 h (60, 61).

Other novel high throughput surveillance techniques have been included in the arsenal of COVID-19 diagnostic testing, including next-generation sequencing (NGS) (15). Swab-Seq, developed by Octant Inc., incorporates a RT-PCR reaction followed by sequencing on Illumina platforms (62). This approach to surveillance testing has been implemented by academic institutions like UCLA (63) and commercial entities like Helix (64). Additionally, tiling approaches for whole genome sequencing of SARS-CoV-2 were optimized by the ARTIC network and others for use with Oxford Nanopore Technologies (ONT) platforms (65–67). These approaches have been adapted to Illumina platforms as well and have found wide application in viral epidemiology. Furthermore, ONT linked a LAMP reaction for viral amplification to a sequencing readout using their long read technology in an assay termed LamPORE™ (68, 69). While NGS has the potential for extremely high throughput, some implementations require 12–24 h to return a result because of the complexities of library preparation and runtime on the instrument. Thus, PCR-based approaches are not intrinsically lower in throughput and, in fact, offer several advantages such as rapid turn-around and quantitative results. In contrast, NGS approaches offer advantages as well. The capacity of NGS to cover the entire viral genome offers improved sensitivity since sub-viral RNA fragments may not be detected in a PCR target. Researchers can also exploit the multiplexing capacity for detecting large panels of respiratory viruses, such as influenza. Lastly, and perhaps most importantly, complete viral sequencing offers the possibility of performing viral epidemiology and analyzing variant spread.

### Limitations

The present study was able to perform a range of assays for SARS-CoV-2 nucleic acid and antibody detection, but there are a number of limitations. First, as part of the study design, the cohort in the present study was a non-random volunteer sample which could be susceptible to selection bias. As such, we may have missed potential SARS-CoV-2 positive participants which could have impacted the evaluation of test methods. However, we feel confident that this would have minimal impact on the present findings given the agreement between the detection of active infection in our cohort compared to reports in Ohio around the time of data collection. Second, the demographics of our cohort did not reflect the demographics of the Dayton, Ohio metropolitan area (70) and the study was conducted over a relatively short time period, limiting the generalizability of the disease presence observed in the present study. Lastly, the small number of active infections did not allow for significant study of the relationship between active infection and antibody kinetics. The participant who supplied the saliva sample with a lower titer was not co-enrolled in the serology aim. While the participant who supplied the saliva sample with the higher titer was co-enrolled in the serology aim, blood/serum samples taken before the positive saliva sample were negative and, as the participants were unenrolled from the study after providing a positive sample, we were unable to follow the relationship of active infection and antibody kinetics.

### Conclusions

This study evaluated the performance of several assays to determine the extent to which COVID-19 was present in a local asymptomatic population situated near a United States Air Force base. Research findings regarding successful testing methodologies both for determining past exposure in the workforce and for detecting active infections can inform return to full capacity planning. Commanders and executives need to make informed decisions about what testing is best for their situation by taking into account a number of factors including prevalence of infection, required sensitivity, desired turnaround time, and available resources. The assays tested here represent only a small sample of SARS-CoV-2 diagnostic and screening tests, and novel techniques are continuously being developed (14, 15, 39, 71). Lessons learned about rapid assay development and deployment during the COVID-19 pandemic will provide insight for future pandemic responses.

## Data Availability Statement

The raw data supporting the conclusions of this article will be made available by the authors, without undue reservation.

## Ethics Statement

The studies involving human participants were reviewed and approved by the Air Force Research Laboratory Institutional Review Board, protocol number FWR20200119H, and conducted in accordance with the provisions of the Belmont Report, Common Rule, and Department of Defense Instruction 3216.02 Protection of Human Subjects guidelines. Written informed consent for participation was not required for this study in accordance with the national legislation and the institutional requirements, however all study participants provided informed consent *via* phone, email, or internet questionnaire prior to enrollment. This manuscript was approved for public release on 15 July 2021; PA case number MSC/PA-2021-0234; AFRL-2021-1853.

## Author Contributions

HP, CH, AI, RJS, RLS and CG contributed to conception and design of the study. RJS, RLS, AMEB, DM, AMB, CD, CM, CS, and CB contributed to acquisition of data. RJS, RLS and MW organized the database. RJS, RLS, AMEB, MW, DM, and CB performed the data analysis. DM, CG, CB, AMB, and CS wrote sections of the manuscript. AMEB, CH, RJS, RLS, HP, and MW prepared the draft of the manuscript. All authors contributed to manuscript revision and approved the submitted version.

## Funding

This study was funded by the Defense Health Agency.

## Conflict of Interest

ABre, DM, CM, CD, and ABra were employed by UES, Inc. The remaining authors declare that the research was conducted in the absence of any commercial or financial relationships that could be construed as a potential conflict of interest.

## Publisher's Note

All claims expressed in this article are solely those of the authors and do not necessarily represent those of their affiliated organizations, or those of the publisher, the editors and the reviewers. Any product that may be evaluated in this article, or claim that may be made by its manufacturer, is not guaranteed or endorsed by the publisher.
